# Early fluid status and severe intraventricular hemorrhage or death in extremely preterm infants

**DOI:** 10.1007/s00467-025-06962-4

**Published:** 2025-09-24

**Authors:** Lucinda J. Weaver, Samuel J. Gentle, Arie Nakhmani, Fazlur Rahman, Namasivayam Ambalavanan, Vivek V. Shukla, Christine Stoops, David Askenazi, Colm P. Travers

**Affiliations:** 1 Department of Pediatrics, University of Alabama at Birmingham, 1700 6Th Avenue South, WIC, Suite 9380, Birmingham, AL 35233 USA; 2 Department of Electrical and Computer Engineering, University of Alabama at Birmingham, Birmingham, AL USA; 3 Department of Biostatistics, University of Alabama at Birmingham, Birmingham, AL USA

**Keywords:** Infant, Premature, Water-electrolyte balance, Hyponatremia, Hypernatremia, Cerebral intraventricular hemorrhage, Mortality

## Abstract

**Background:**

Measures of early postnatal fluid balance may be associated with severe intraventricular hemorrhage (sIVH) and/or death in extremely preterm infants in the first postnatal week.

**Methods:**

A single-center, retrospective cohort study including actively treated inborn infants weighing ≥ 400 g and 22–27 weeks’ gestation from 2014–2021. Longitudinal mixed effect models compared daily fluid balance covariates including serum sodium, percent weight change, total fluid intake, urine output, and fluid balance (daily weight – birth weight /birth weight × 100) among infants with and without sIVH or death, during the first seven postnatal days. Multiple regression and machine learning models were developed to predict sIVH and/or death. Variables that were incorporated into the models included measures of fluid balance, gestational age, birth weight, antenatal corticosteroids, multiples, and sex.

**Results:**

We included 932 infants with mean ± SD gestational age of 25w2d ± 11d and birth weight of 746 ± 212 g of whom 195 (20.9%) had sIVH and/or death. Lower percentage weight change (*p* < 0.001), higher total fluid intake (*p* = 0.007), higher sodium (*p* = 0.007), and positive early fluid balance (*p* < 0.001) were associated with sIVH and/or death even after adjustment for baseline characteristics. The area under the receiver-operating curve (AUC) for regression models predicting sIVH and/or death incorporating baseline characteristics improved after adding fluid balance measures from 0.75 to 0.80, while the AUC for machine learning models improved from 0.72 to 0.84.

**Conclusions:**

In extremely preterm infants, early fluid status measures were associated with risk of sIVH and/or death. The addition of fluid status measures improves the performance of models predicting sIVH and/or death.

**Graphical abstract:**

**Figure:**
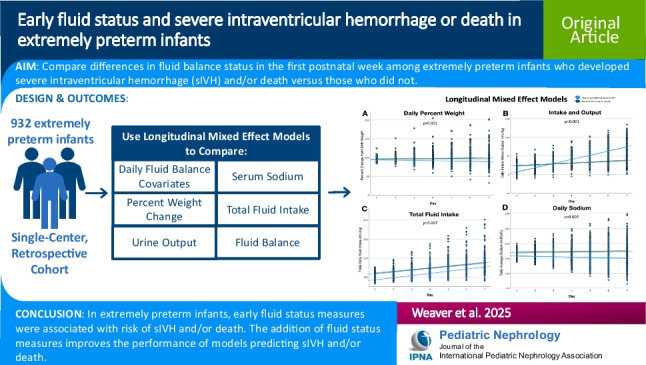
A higher resolution version of the Graphical abstract is available as Supplementary information

**Supplementary Information:**

The online version contains supplementary material available at 10.1007/s00467-025-06962-4.

## Introduction

Extremely preterm infants are at high risk of developing severe intraventricular hemorrhage (sIVH) which is associated with a significantly higher risk of mortality and morbidity including adverse neurodevelopmental outcomes [1–3]. Perinatal risk factors associated with intraventricular hemorrhage include low birth weight, gestational age, sex, absence of antenatal corticosteroids, intrauterine infection, and breech delivery [4, 5]. Hemodynamic fluctuations and disturbances in fluid balance are common in the first 72 postnatal hours. During the first postnatal week, weight loss due to diuresis with natriuresis is part of extrauterine adaptation [6]. Extremely preterm infants are at high risk of inadequate extrauterine adaptation because they have significant insensible fluid losses, in addition to immature mechanisms to maintain homeostatic balance [7–10] and are therefore reliant on parenteral fluids to maintain their fluid and electrolyte balance.

Clinicians typically commence parenteral fluids containing minimal to no sodium after birth and then follow trends in sodium levels, weight changes, and urine output to determine changes to the total fluid intake [11]. The optimal fluid balance targets for percent weight loss, serum sodium, and total fluid intake are controversial. Randomized clinical trials suggest that liberal early fluid administration increases the risk of patent ductus arteriosus in preterm infants, necrotizing enterocolitis, and death, while rates of IVH and bronchopulmonary dysplasia did not differ significantly [12, 13]. An association between dysnatremia and the development of IVH and adverse neurodevelopmental outcomes has been reported [4, 5, 14–16]. Dysnatremia could be a surrogate measure for fluid balance status [17]. Early fluid balance and dysnatremia might represent modifiable postnatal therapies that could be addressed to improve outcomes of extremely preterm infants.

The primary aim of this study was to compare differences in fluid balance status in the first postnatal week among extremely preterm infants who developed sIVH and/or death versus those that did not. The secondary aim was to evaluate if the addition of fluid balance metrics into multivariable prediction models enhanced the prediction of sIVH or death. We hypothesized that higher total fluid intake, dysnatremia, lower urine output, and excessive or inadequate weight loss in the first postnatal week would be independently associated with sIVH or death.

## Methods

This single-center, retrospective cohort study included actively treated infants with birth weight ≥ 400 g and gestational age from 22^0/7^ to 27^6/7^ weeks born at the University of Alabama at Birmingham from April 2014 to December 2021 [18]. Infants with major congenital anomalies including complex congenital heart disease, suspected or confirmed syndromes, suspected or confirmed severe metabolic disease, and/or severe bilateral congenital kidney or urogenital disease were excluded. Perinatal characteristics included baseline maternal demographic information, gestational age, sex, race and ethnicity as determined by maternal self-report, mode of delivery, multiples, Apgar scores at 5 min, and receipt of antenatal corticosteroids.

Day of birth was defined as postnatal day one. Postnatal information was collected through the 7th postnatal day in 24-h intervals including daily weight, daily percent weight change from birth weight, peak percent weight loss (negative) or gain (positive) from birth weight, daily total fluid intake (TFI) in milliliters per kilogram (mL/kg) based on birth weight, and daily urine output (UOP) in mL/kg/hour based on birth weight. Laboratory analyses including average daily serum sodium level, first hematocrit, and packed red blood cell transfusion volume in the first seven days of life were collected from the electronic health record. Total daily fluid intake (mL/kg/d) was calculated including parenteral fluid administration, medications, flushes, feedings, blood products and boluses in a 24 h period. Determination of fluid balance was calculated using the following equation: fluid balance = (daily weight—birth weight/birth weight) × 100. This method may provide the best surrogate for daily fluid balance status in this population and is consistent with previous studies [7–10].

Data collected through the time of discharge included neonatal morbidities as defined by the Eunice Kennedy Shriver National Institute of Child Health and Human Development Neonatal Research Network: bronchopulmonary dysplasia grade ≥ 2 at 36 weeks’ postmenstrual age [19], hemodynamically significant patent ductus arteriosus, necrotizing enterocolitis ≥ grade 2 as defined by modified Bell’s Criteria, spontaneous intestinal perforation, and death in the first postnatal week or prior to discharge. This study was approved by the institutional review board at the University of Alabama at Birmingham with a waiver of informed consent (Institutional Review Board approval number IRB-150908001). This analysis followed the transparent reporting of a multivariable prediction model for individual prognosis or diagnosis (TRIPOD) reporting guidelines [20].

All infants were managed per standardized unit guidelines for initial fluid administration and incubator humidification [18]. On postnatal day one, the total fluid intake was restricted to approximately 70–90 mL/kg/day including 10% dextrose solution with 10% amino acid solution, medications, flushes, and heparinized arterial fluid containing half-normal sodium acetate (if applicable). Daily total fluid intake was increased by 10–20 mL/kg/day per clinical team’s discretion typically based on percent weight change, urine output, and the serum sodium. Enteral intake was included in the overall total fluid intake amount. Infants were managed in an incubator with 80% humidity from birth, which was typically decreased to 60% on postnatal day 4, then to 50% by postnatal day 5–7.

The primary outcome was sIVH, defined as grade 3 or 4 by the Papile Grading System, and/or death within the first seven postnatal days [3]. Death was included in the composite outcome as some infants may have died before a head ultrasound was done or because of clinical instability from sIVH. Data were analyzed using the Student’s t-test or the Mann–Whitney test for continuous variables and the chi-square test or Fisher’s exact test for the categorical variables. Longitudinal mixed effect models were used to compare trends from ordered predictors by treating these variables as continuous covariates to evaluate fluid balance covariates throughout the first postnatal week among infants with and without sIVH and/or death. Odds ratios (ORs) and associated 95% confidence intervals (CI) for the association between fluid balance variables and the primary outcome were estimated from logistic regression models adjusted for baseline characteristics including gestational age, birth weight, antenatal corticosteroids, multiples, and sex to account for potential confounding [21].

Multivariable logistic regression models and machine learning models were developed, adjusting for baseline characteristics, including gestational age, birth weight, antenatal corticosteroids, multiples, and sex with and without fluid balance measures to assess the prediction accuracy for sIVH or death. The Kruskal–Wallis feature-ranking algorithm was used to identify the fluid balance covariates predictive of sIVH and/or death in the first postnatal week for extremely preterm infants. Machine learning classifiers including Logistic Regression, Naïve Bayes Classifier, and Ensemble RUS Boosted Trees were used to incorporate the five underlying demographics with or without the top 20 fluid balance covariates into the model. Missing data were not imputed into models and a sensitivity analysis was conducted including data for the initial three postnatal days as there were relatively few infants (approximately 5%) who died and had missing data within the first 72 h after birth. The accuracy of all models was assessed using the area under the receiver operating curves (AUC). In all analyses, a 2-tailed *p* < 0.05 was considered statistically significant.

## Results

A total of 932 infants were included in the analysis. The mean gestational age of infants was 25w2d ± 1w4d and the average birth weight was 746 ± 212 g. There were a total of 195 infants (20.9%) with sIVH and/or death within the first postnatal week. The rates of sIVH, death, or combined were 13.5% (*n* = 126), 9.8% (*n* = 91), and 2.3% (*n* = 22), respectively. Infants with sIVH and/or death had a lower gestational age (24w ± 1.6 versus 26w ± 1.4; *p* < 0.001) and birth weight (621 ± 176 g versus 779 ± 209 g; *p* < 0.001). These infants were less likely to be born via cesarean section and more likely to be breech vaginal births, Black, and born with lower Apgar scores, have higher initial hematocrit (43 ± 6% versus 40 ± 7%; *p* < 0.001), and receive a higher volume of transfusions during the first week (23 ± 63 mL/kg versus 76 ± 101 mL/kg, *p* < 0.001). There was no difference in sex, rates of administration of antenatal corticosteroids, or multiple gestation (Table 1).

**Table 1:** Comparison of maternal and neonatal characteristics between infants who survived without severe intraventricular hemorrhage (IVH) and infants with severe IVH and/or death within the first postnatal week

Characteristics	Survived w/o Severe IVH (*n* = 737)	Severe IVH and/or Death (*n* = 195)	*P* value
Female, *n* (%)	390 (52.9)	93 (47.7)	0.26
White, *n* (%)	275 (37.3)	58 (29.7)	0.05
Black, *n* (%)	411 (55.8)	127 (65.1)	
Hispanic, *n* (%)	28 (3.8)	4 (2.0)	
Unknown/Other, *n* (%)	23 (3.1)	6 (3.1)	
Birth weight, mean ± SD, (grams)	779 ± 209 g	621 ± 176 g	< 0.001
Gestational age, mean ± SD (weeks and days)	25w4d ± 1w4d	24w1d ± 1w4d	< 0.001
22 wga (%)	28 (3.8)	40 (20.5)	
23 wga (%)	96 (13.0)	54 (27.7)	
24 wga (%)	98 (13.3)	40 (20.5)	
25 wga (%)	128 (17.4)	20 (10.3)	
26wga (%)	177 (24.0)	27 (13.8)	
27 wga (%)	210 (28.5)	14 (7.2)	
Antenatal corticosteroids, *n* (%)	647 (88.0)	166 (85.1)	0.40
Multiple gestation, *n* (%)	166 (22.5)	53 (27.2)	0.18
Cesarean section, *n* (%)	471 (64.0)	92 (47.2)	< 0.001
Vaginal Delivery—vertex, *n* (%)	240 (32.6)	79 (40.5)	
Vaginal Delivery—breech, *n* (%)	24 (3.2)	24 (12.3)	
Apgar Score at 5 min, median (IQR)	6 (5–7)	4 (2–6)	< 0.001
Initial Hematocrit, mean ± SD (%)	43 ± 6	40 ± 7	< 0.001
Volume of transfusions, mean ± SD (mL/kg)	23 ± 63	76 ± 101	< 0.001

**Fig. 1:**
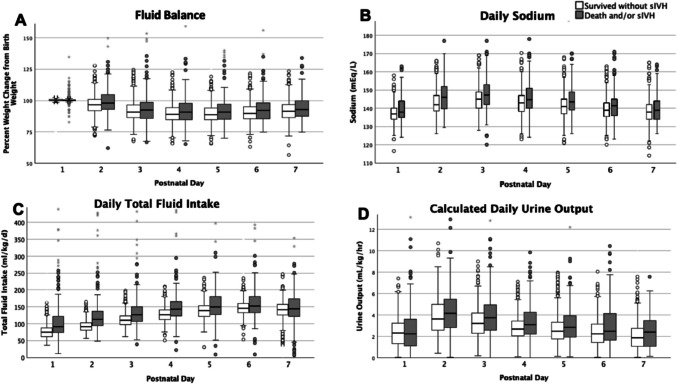
Boxplots of fluid balance and covariates throughout the first postnatal week in extremely preterm infants. Boxplots within each panel represent the covariates of infants with and without severe intraventricular hemorrhage (sIVH) and/or death within the first postnatal week. The boxplots represent the fluid balance calculated as (daily weight/birth weight × 100) (**A** fluid balance), average daily sodium (mEq/L) (**B** daily sodium), daily total fluid intake (mL/kg/d) calculated with total fluid intake in a 24-h period divided by birth weight (**C** daily total fluid intake), and adjusted daily urine output (ml/kg/hr) calculated as total urine output in a 24-h period divided by birth weight and 24 h (**D** daily calculated urine output)

**Fig. 2:**
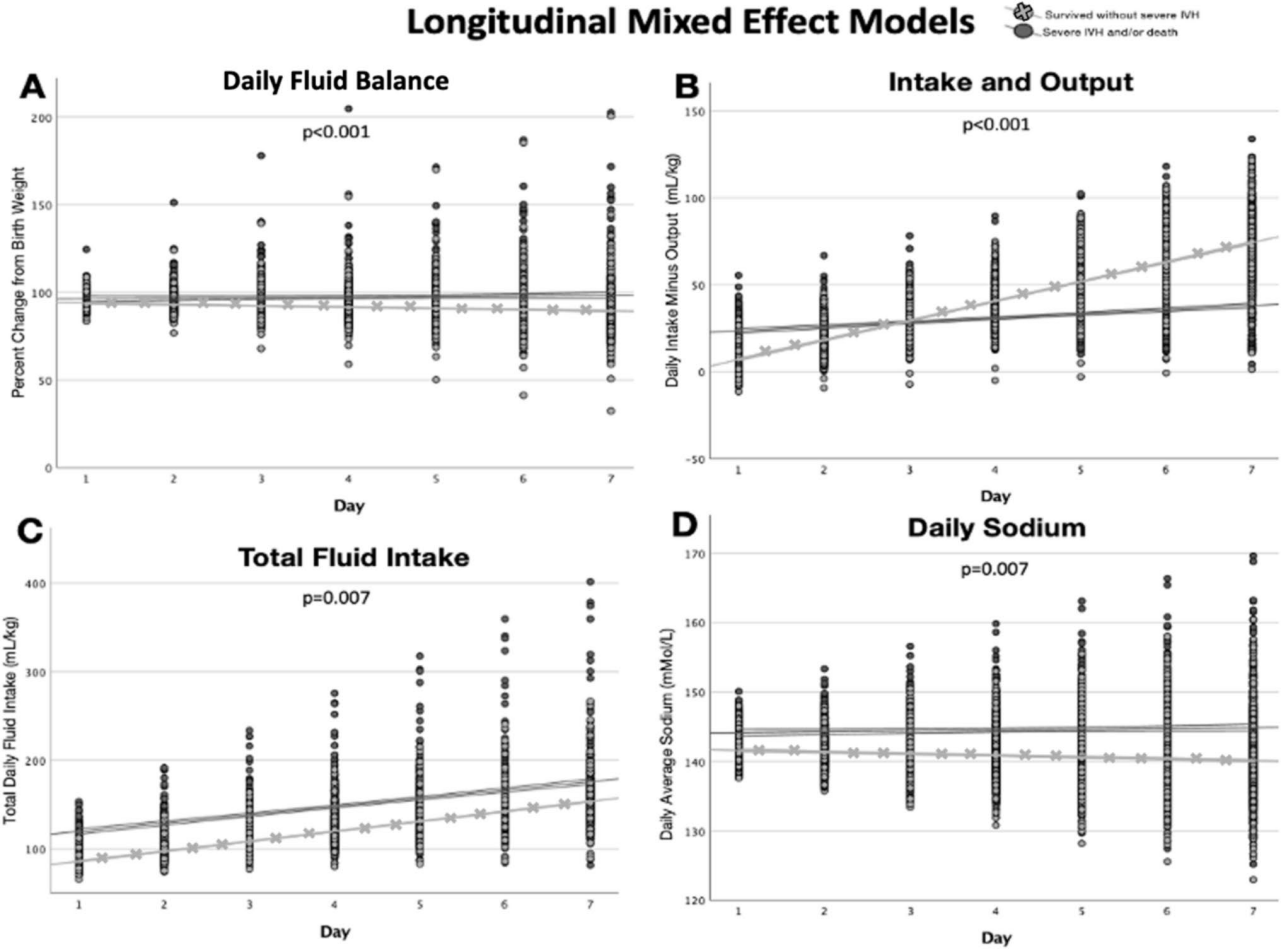
Longitudinal mixed effect models for fluid balance covariates throughout the first postnatal week in extremely preterm infants. Linear regression lines within each panel represent changes in covariates of infants with and without severe intraventricular hemorrhage (IVH) and/or death. Panels indicate the following changes over time: daily weight percent change from birth weight (daily weight/birth weight × 100) (daily fluid balance (**A**), *P* < 0.001), difference between total intake fluid and output (intake and output (**B**), *P* < 0.001), total daily fluid intake (**C**, *P* = 0.007), and daily average sodium (sodium (**D**), *P* = 0.007)

The median negative peak fluid balance of the entire cohort in the first postnatal week was −13.4% (IQR, −17.8 to −8.2%). Infants with sIVH and/or death had a median peak negative fluid balance of −10.6% (IQR −17.3 to 0.0%) compared with −13.7% (IQR −18.1 to −9.3%) among infants who survived without sIVH (Fig. 1, Supplemental Table 1). Among infants with a positive peak fluid balance, infants with sIVH and/or death peaked at a median of 2.0% (IQR, 0.0 to 9.4%) and infants who survived without sIVH peaked at a median of 0.2% (IQR, 0.0 to 4.4%) within the first week after birth (Fig. 1, Table 2, Supplemental Table 1). Adjusted longitudinal mixed effect models found a lower peak fluid balance was associated with sIVH and/or death in the first postnatal week (*p* < 0.001) (Fig. 2).

**Table 2:** Fluid balance measures among extremely preterm infants that survived without severe intraventricular hemorrhage (sIVH) and infants with sIVH and/or death within the first postnatal week. Total fluid intake is represented for postnatal day 2 and was the most significant factor for prediction of sIVH and/or death based on multiple regression models. The fluid balance as calculated (daily weight − birth weight/birth weight) × 100 was most reflective of the total fluid intake on postnatal day 2

Outcomes	Survived w/o severe IVH (*n* = 736)	Severe IVH and/or death (*n* = 195)	*P* value
Peak negative fluid balance, median % (IQR)	− 13.7 (− 18.1 to − 9.3)	− 10.6 (− 17.3 to 0)	< 0.001
Peak positive fluid balance, median % (IQR)	0.2 (0 to 4.4)	2.0 (0 to 9.4)	< 0.001
Total fluid intake postnatal day 2 (ml/kg/d), median (IQR)	95 (81 to 103)	112 (95 to 136)	0.019
Fluid balance on postnatal day 3, median (IQR)	− 9.3 (− 13.6 to − 3.5)	− 7.5 (− 14.0 to − 1.5)	0.017
Average daily sodium (mmol/l) (IQR)	141 (138 to 143)	144 (139 to 149)	< 0.001

A higher average daily sodium was associated with sIVH and/or death (*p* = 0.007) (Fig. 2) with a median of 145 mmol/L in those with sIVH and/or death compared with 140 mmol/L in those who survived without sIVH (Fig. 1, Table 2, Supplemental Table 1). Conversely, higher total fluid intake was associated also with sIVH and/or death in the first postnatal week (*p* = 0.007) (Fig. 1, Fig. 2, Supplemental Table 1). Infants with sIVH and/or death received on average, 15–20 mL/kg/d more fluid over the first week (Table 2, Fig. 2). Daily urine output was not statistically significantly different between groups (*p* = 0.08) (Fig. 1, Fig. 2, Supplemental Table 1). Those with sIVH and/or death initially had a positive early fluid balance which remained positive but relatively flat over the first week whereas those who survived without sIVH initially had a more even initial fluid balance which gradually increased over the first week (*p* < 0.001) (Fig. 2).

**Fig. 3:**
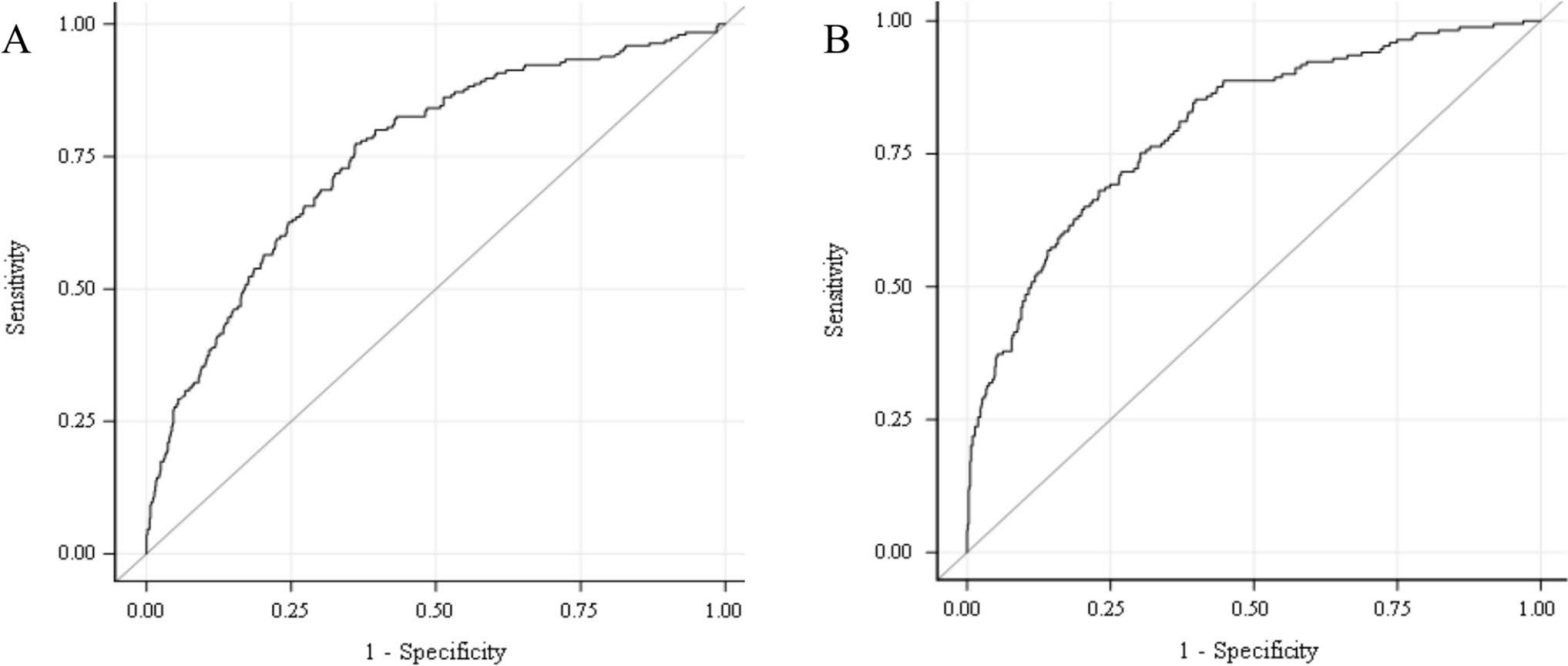
Multivariate logistic regression models for prediction of severe IVH and/or death in extremely preterm infants. Using multivariate logistic regression models incorporating the key baseline covariates (gestational age, birth weight, sex, antenatal corticosteroids, and multiples), the area under the receiver operating curve (AUC) was 0.75 (**A**). Univariate logistic regression with unadjusted odds ratios with 95% confidence limits was used to determine the association with individual available fluid balance status predictors. Total fluid intake on day 2 was the top predictor, and the AUC was increased to 0.80 by adding this covariate alone (**B**). The addition of other fluid covariates did not improve the model further

Regression models incorporating baseline characteristics predicting sIVH and/or death had an AUC = 0.75. With the addition of fluid balance measures the AUC improved to 0.80 (Fig. 3). The TFI on postnatal day 2 was the most statistically significant fluid covariate in the multiple regression models, with every increase in 1 unit of TFI (mL/kg/day) associated with a 2% higher risk of sIVH and/or death. TFI on postnatal day 2 was 95 ml/kg/day (81 to 103) for infants who survived without sIVH in comparison to 112 ml/kg/d (95 to 136) with sIVH and/or death (*p* = 0.019) (Table 2).

**Fig. 4:**
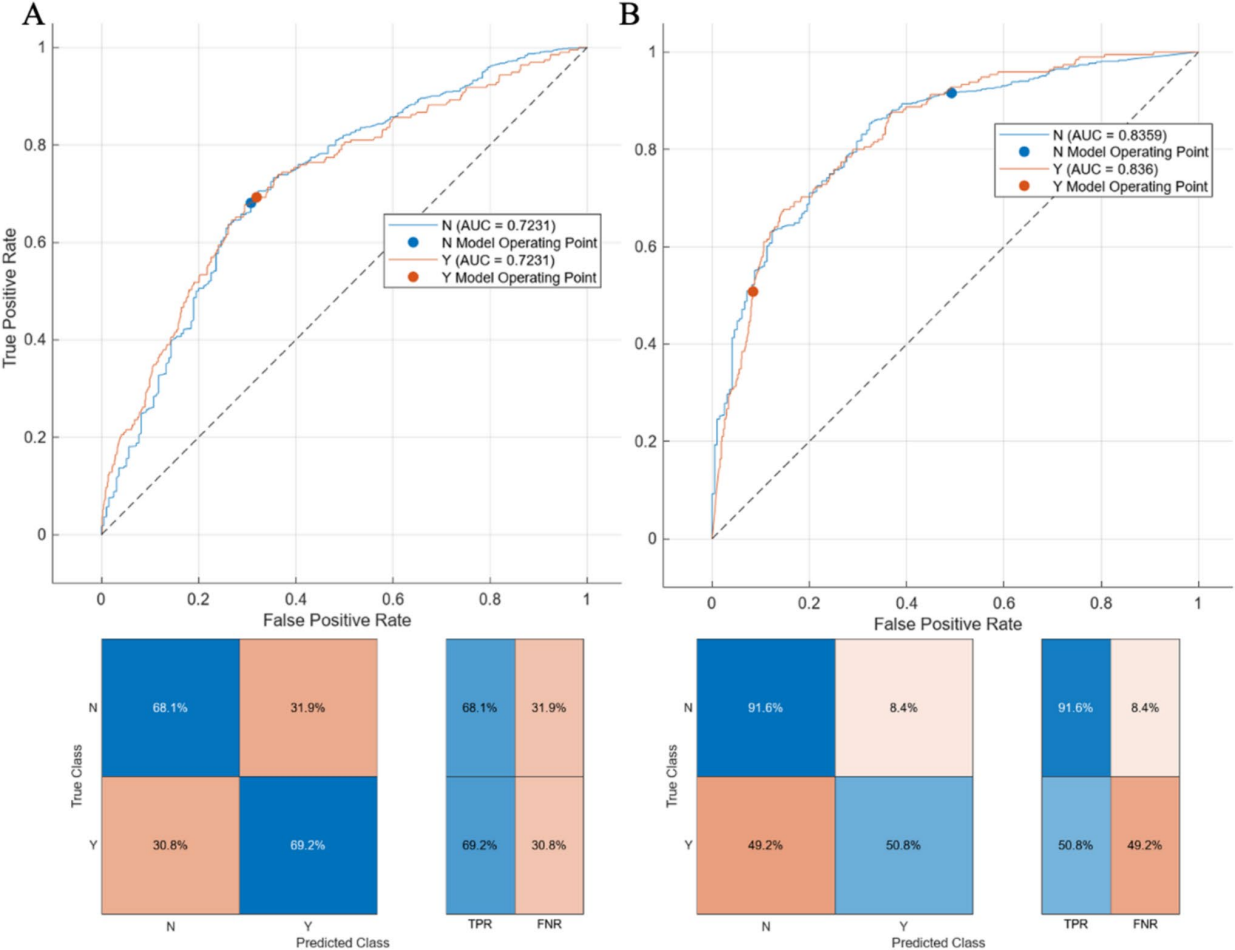
Machine learning models for the prediction of severe IVH or death in extremely preterm infants. Using ensemble boosted tree models incorporating the key baseline covariates (gestational age, birth weight, sex, antenatal corticosteroids, and multiple) the area under the receiver operating curve (AUC) was 0.72 (**A** accuracy 68.3%). Kruskal–Wallis feature ranking algorithm was used to identify the fluid balance covariates predictive of severe IVH and/or death in the first postnatal week for extremely preterm infants. Using Naïve Bayes classifier, the AUC improved to 0.84 with the incorporation of the top 20 fluid balance covariates (**B** accuracy 83.0%)

After inclusion of total fluid intake on day 2, the addition of other fluid covariates did not significantly improve the regression model performance. Total fluid intake on postnatal day 2 was the top individual predictor for sIVH and/or death (OR 1.022, CI 1.015–1.029, *p* < 0.001) in regression models and was consistently selected as a top feature using the Kruskal–Wallis feature ranking algorithm for the machine learning models. Naïve Bayes Classifier was the most accurate ML-based model for sIVH and/or death. Naïve Bayes Classifier incorporating baseline characteristics improved from 0.72 to 0.84 with the addition of fluid balance measures for the top 20 most predictive fluid balance covariates in the first postnatal week (Fig. 4, Supplemental Table 2). In a sensitivity analysis including the top 20 most predictive fluid balance covariates from only the first 3 postnatal days, the AUC for prediction of sIVH and/or death was 0.88.

## Discussion

In this single-center, retrospective cohort study of extremely preterm infants we report the association of fluid balance measures with sIVH and/or death in the first postnatal week. In addition, the accuracy of conventional and machine learning models was improved with the addition of fluid balance measures. Infants with sIVH and/or death lost less weight, received more fluid, and had a higher sodium in comparison to those who survived without sIVH. The combination of decreased postnatal weight loss and increased fluid intake, but with a higher serum sodium suggests that these infants were fluid overloaded in combination with a higher sodium intake (sufficient to overcome the dilution factor of higher total body water). Fluid balance measures may be a modifiable predictor of sIVH and/or death in extremely preterm infants. Close monitoring of fluid balance and fluid restriction in the first postnatal days may represent an early management strategy that could reduce risk of sIVH.

Studies assessing fluid balance with outcomes in premature infants have mostly focused on respiratory outcomes [7, 8, 10, 22, 23]. Relatively few studies have evaluated the association between fluid balance and sIVH. Our study is consistent with a single center retrospective analysis of 210 extremely low birth weight infants, which reported that higher total fluid intake (99 ± 32 versus 83 ± 16 ml/kg/day; *p* = 0.011) and sodium intake (2.3 ± 2.3 versus 0.5 ± 1.0 mEq/kg/day; *p* < 0.001) on day 2 were associated with sIVH [5]. That study also suggested that the association between higher TFI in the first 4 postnatal days and sIVH was at least in part related to a higher number of transfusions among infants with sIVH [5]. In addition to transfusions leading to a higher TFI, it is also possible that the sodium contained in blood products contributes to differences in rates of sIVH, even in the absence of dysnatremia [24]. Furthermore, the higher sodium could also be due to higher sodium intake in response to sIVH, as metabolic acidosis and more transfusions are associated with sIVH and underperfusion may lead clinicians to supplement sodium acetate or bicarbonate and blood products, thereby increasing the sodium load.

In the current study fluid balance was associated with the development of severe IVH. Additionally, fluid balance is significantly affected by kidney function. Acute kidney injury (AKI) has been independently associated with IVH but the timing of IVH in relation to AKI is not clear. In the current study, we did not have data on the timing of sIVH and recent data suggest that approximately 50% may occur after 48 h [25]. Due to a lack of complete creatinine data in the first postnatal week in this retrospective study we were unable to accurately assess AKI for all infants in our study. AKI may account for more significant fluid overload in infants with severe IVH or death but this was not captured in our study. Changes in blood pressure and the use of vasopressors have been associated with sIVH or death although placebo-controlled randomized clinical trials of vasopressors have not demonstrated a difference [26, 27]. Trials of vasopressors in the context of poor perfusion suggest a potential clinically important difference in IVH although the difference was not significant [28]. We did not collect prospective data on perfusion, boluses, and vasopressors in this retrospective cohort study and we were unable to determine the exact indication for treatments used for hypotension. We did not directly collect data on measures of perinatal asphyxia that might be associated with sIVH but we noted a lower Apgar score at 5 min in infants with severe IVH or death, which may be collinear with perinatal asphyxia [29].

In a secondary analysis of the Preterm Erythropoietin Neuroprotection Trial (PENUT), extremely preterm infants with a lower peak negative weight loss on day 3 and higher peak positive weight gain in the first 2 weeks were more likely to require mechanical ventilation at postnatal day 14 [10]. IVH was included as a secondary outcome and infants with less weight loss or more weight gain also had a higher rate of IVH (47.6% versus 23.7%, *p* < 0.001). In another secondary analysis of the PENUT trial evaluating total fluid intake and maximal weight loss in the first postnatal week, it was noted that infants with higher total fluid intake had a higher risk of PDA and NEC but there was no difference in IVH (all stages). In a multicenter retrospective cohort study of 9275 preterm infants it was noted that there was a “U”-shaped relationship between weight change from birth to postnatal day 3 and death or severe brain injury [25]. In our study, a higher peak percent weight loss and a lower total fluid intake were both associated with a lower risk of sIVH and/or death. Although we had few missing data, we noted that restricting our data to the first 3 postnatal days improved our prediction of sIVH and/or death which was likely due to the first 3 postnatal days being the most influential as related to sIVH in addition to a reduction of noise from data of infants with missing or incomplete data beyond 72 h after birth.

## Limitations

All findings in our study represent associations due to the retrospective nature of the analysis. As the exact timing of IVH was not known, it is possible that changes in fluid balance measures may be a secondary result rather than causal. Despite efforts to control for known risk factors for the development of sIVH and death, we were unable to control for all possible confounding variables that may remain. All infants studied were from a single center, tertiary unit who were treated with a standardized protocol which includes fluid restriction with no to minimal sodium intake and highly humidified incubators for the first 72 h after birth. While this standardization allows for a relatively homogeneous cohort for analysis, it does not account for practice variation across centers and our results require external validation. This study included all fluid administration to these infants including blood products, saline boluses, medications, and flushes which provides a realistic evaluation of the fluid balance in these infants. However, the parenteral and enteral fluid administration was not differentiated and could be a confounding variable [26].

## Conclusions

Fluid balance is associated with sIVH and/or death in extremely preterm infants. The results of this study support judicious early fluid administration and avoidance of severe hypernatremia while awaiting diuresis and natriuresis to lower the risk of sIVH and/or death in these vulnerable infants.

## Supplementary Information

Below is the link to the electronic supplementary material.

Graphical abstract (PPTX 356 KB)

ESM 2 (DOCX 18.4 KB)
