# Supplementary material for: Early fluid status and severe intraventricular hemorrhage or death in extremely preterm infants
Source: Pediatr Nephrol. 2025 Sep 24;41(1):239–47. doi: 10.1007/s00467-025-06962-4 (PMC12685979; doi:10.1007/s00467-025-06962-4)
Supplement: Supplementary file 1 — Graphical abstract (PPTX 356 KB) [file 467_2025_6962_MOESM1_ESM.pptx]

## Slide 1
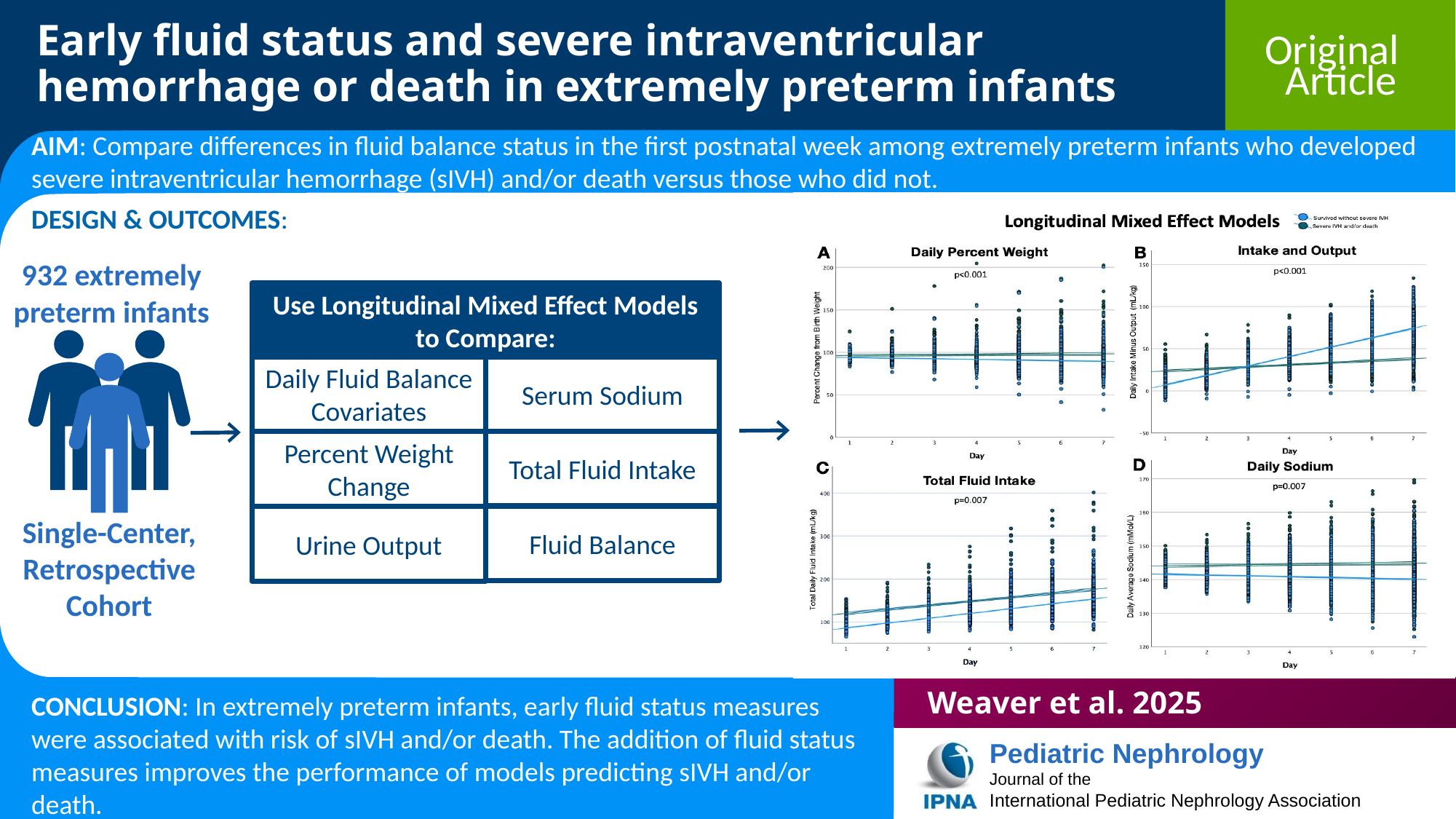

Early fluid status and severe intraventricular hemorrhage or death in extremely preterm infants
AIM: Compare differences in fluid balance status in the first postnatal week among extremely preterm infants who developed severe intraventricular hemorrhage (sIVH) and/or death versus those who did not.
DESIGN & OUTCOMES:
932 extremely preterm infants
Single-Center,
Retrospective
Cohort
Use Longitudinal Mixed Effect Models to Compare:
Daily Fluid Balance Covariates
Serum Sodium
Total Fluid Intake
Percent Weight Change
Fluid Balance
Urine Output
Weaver et al. 2025
CONCLUSION: In extremely preterm infants, early fluid status measures were associated with risk of sIVH and/or death. The addition of fluid status measures improves the performance of models predicting sIVH and/or death.
