# Supplementary material for: Early fluid status and severe intraventricular hemorrhage or death in extremely preterm infants
Source: Pediatr Nephrol. 2025 Sep 24;41(1):239–47. doi: 10.1007/s00467-025-06962-4 (PMC12685979; doi:10.1007/s00467-025-06962-4)
Supplement: Supplementary file 2 — ESM 2 (DOCX 18.4 KB) [file 467_2025_6962_MOESM2_ESM.docx]

Supplemental Table 1: Data of fluid balance covariates by outcomes throughout the first postnatal week in extremely preterm infants. Data represent the covariates of infants with and without severe intraventricular hemorrhage (sIVH) and/or death within the first postnatal week. The fluid balance calculated as (daily weight/birth weight X 100), average daily sodium (mEq/L), daily total fluid intake (mL/kg/d) calculated with total fluid intake in a 24 hour period divided by birth weight, and adjusted daily urine output (ml/kg/hr) calculated as total urine output in a 24 hour period divided by birth weight and 24 hours.

| Fluid Covariates | Outcome | Postnatal Day 1 | Postnatal Day 2 | Postnatal Day 3 | Postnatal Day 4 | Postnatal Day 5 | Postnatal Day 6 | Postnatal Day 7 |
| --- | --- | --- | --- | --- | --- | --- | --- | --- |
| Mean Fluid Balance  (% of birth weight) ± SD | Survived without sIVH | 100.3 ± 5.7 | 96.7 ± 8.9 | 90.7 ± 9.2 | 90.0 ± 9.1 | 89.9 ± 9.0 | 90.8 ± 8.7 | 92.3 ± 9.0 |
|  | sIVH or Death | 101.1 ± 5.0 | 99.4 ± 11.5 | 92.5 ± 14.9 | 93.7 ± 16.5 | 94.0 ± 17.0 | 95.9 ± 19.4 | 95.9 ± 13.0 |
| Median Daily Sodium  (mEq/L)  (IQR) | Survived without sIVH | 137 (134 to 140) | 143 (139 to 147) | 145 (140 to 149) | 143 (139 to 147) | 141 (137 to 145) | 140 (135 to 143) | 138 (138 to 142) |
|  | sIVH or Death | 139 (35 to 144) | 146 (140 to 152) | 147 (142 to 153) | 146 (140 to 151) | 145 (139 to 149) | 142 (136 to 145) | 140 (135 to 145) |
| Mean Daily Total Fluid Intake  (mL/Kg/d) ± SD | Survived without sIVH | 78.4 ± 22.4 | 94.9 ± 21.8 | 111.6 ± 24.6 | 128.4 ± 24.7 | 139.0 ± 27.0 | 148.8 ± 32.0 | 137.8 ± 34.9 |
|  | sIVH or Death | 89.5 ± 30.3 | 115.7 ± 52.5 | 122.7 ± 31.4 | 143.4 ± 44.8 | 147.6 ± 35.9 | 156.0 ± 42.9 | 142.5 ± 63.0 |
| Mean Daily Urine Output  (mL/Kg/24hrs)  ± SD | Survived without sIVH | 2.4 ± 1.4 | 3.8 ± 1.7 | 3.3 ± 1.4 | 2.8 ± 1.3 | 2.7 ± 1.4 | 2.4 ± 1.3 | 2.1 ± 1.4 |
|  | sIVH or Death | 2.7 ± 2.2 | 4.3 ± 2.2 | 3.7 ± 2.1 | 3.5 ± 2.0 | 3.2 ± 1.9 | 3.0 ± 2.1 | 2.6 ± 2.1 |

| **Ranking** | **Fluid Balance Covariates** | **Mean Rank** |
| --- | --- | --- |
| 1 | Gestational Age | 52.28 |
| 2 | Total Fluid Intake (mL/kg/d) Day 2 | 51.80 |
| 3 | Birth Weight (kg) | 49.48 |
| 4 | Weight (g) Day 1 | 47.16 |
| 5 | Apgar 5 mins | 41.29 |
| 6 | Weight (g) Day 2 | 32.61 |
| 7 | Total Fluid Intake (mL/kg/d) Day 1 | 32.31 |
| 8 | Weight Day 3 | 28.36 |
| 9 | Weight Day 4 | 27.66 |
| 10 | Total Fluid Intake (mL/kg/d) Day 4 | 25.56 |
| 11 | Total Fluid Intake (mL/kg/d) Day 3 | 25.25 |
| 12 | Weight (g) Day 5 | 25.09 |
| 13 | Weight (g) Day 7 | 20.07 |
| 14 | Weight (g) Day 6 | 19.93 |
| 15 | Sodium Day 2 | 15.42 |
| 16 | Total Intake (mL/d) Day 6 | 13.09 |
| 17 | Sodium Day 5 | 12.82 |
| 18 | Sodium Day 3 | 12.13 |
| 19 | Total Intake (mL/d) Day 7 | 11.52 |
| 20 | Total Intake (mL/d) Day 5 | 11.02 |

Supplemental Table 2: Fluid balance covariates predictive of extremely preterm infants that survived without severe intraventricular hemorrhage (sIVH) and infants with sIVH and/or death within the first postnatal week ranked based on Kruskal Wallis feature-ranking algorithm.
